# Evaluating the Efficacy of Common Femoral Endarterectomy With Profundoplasty: A Three-Year Single-Center Perspective

**DOI:** 10.7759/cureus.74809

**Published:** 2024-11-30

**Authors:** Hisham Abdelrheem Abdelrhman Hamad, Reyad ABD Allah, Sohaib Jararaa, Mohamed S M Elshikhawoda, Omer Kamal Omer, Muhammad Awais Zafar, Yara Ibrahim, Mahmoud Okaz, Mohammed Jibreel Mohammed, Tarig Barakat

**Affiliations:** 1 Surgery, University of Bahri, Khartoum, SDN; 2 Plastic Surgery, Khartoum North Hospital, Khartoum, SDN; 3 Vascular Surgery, Glan Clwyd Hospital, Rhyl, GBR; 4 Orthopedic Surgery, Luton and Dunstable Hospital NHS Foundation Trust, London, GBR; 5 Vascular Surgery, Betsi Cadwaladr University Health Board, Wrexham, GBR; 6 General Surgery, Betsi Cadwaladr University Health Board, Wrexham, GBR; 7 General and Colorectal Surgery, University Hospital Wishaw, Glasgow, GBR; 8 Vascular and Endovascular Surgery, Glan Clwyd Hospital, Rhyl, GBR

**Keywords:** clti, common femoral endarterectomy, iliofemoral occlusive disease, profundoplasty, rest pain

## Abstract

Background: This study aims to synthesise recent findings on the outcomes of common femoral endarterectomy (CFE) with profundoplasty, evaluating the efficacy, complications, and predictors of long-term success in patients undergoing this procedure.

Patients and methods: This is a descriptive retrospective study assessing the outcomes of CFE with profundoplasty. All patients with chronic limb-threatening ischaemia (CLTI) who attended and underwent CFE with profundoplasty with or without iliac intervention at Glan Clwyd Hospital (Wales, United Kingdom) were studied. We excluded the patients who had the CFE as part of bypass surgery.

Results: In a period of three years, 77 patients were included. Forty-six (59.7%) of them were male, and 31 (40.3%) of them were female. The mean age was 73.3 ± 7.8 years (standard deviation). Regarding the comorbidity, hypertension and smoking were recorded for most of the patients, while the other comorbidities were less frequent. The majority of the patients are presented with rest pain 70.1% (n = 54), while the remainder has both rest pain and tissue loss. Forty patients (51.9%) had CFE as part of the hybrid operation with iliac angioplasty; however, only 37 patients (48.1%) had CFE. The most reported complications postoperatively were groin infection, hospital-acquired pneumonia, and thrombosis, with less reporting for the rest of the complications. The limb salvage rate was 74% (n = 57); moreover, the mean primary patency rate was 10.7 + 8.4 months SD.

Conclusion: The CFE is a feasible operation, either isolated or in conjunction with iliac angioplasty, with a satisfactory patency rate. It can be offered to the high-risk group of patients. However, they are going to need a rigorous follow-up to avoid or minimise postoperative complications.

## Introduction

Common femoral endarterectomy (CFE) is a surgical intervention designed to treat atherosclerotic disease in the common femoral artery (CFA), an essential artery in the circulatory supply of the lower extremities. Given the increasing incidence of peripheral artery disease (PAD), comprehending the outcomes of CFE is crucial for enhancing patient treatment and informing surgical decisions. Research indicates that individuals with considerable stenosis in the CFA may benefit from this technique, which seeks to relieve symptoms such as claudication, enhance limb perfusion, and diminish the risk of limb loss and cardiovascular incidents [1,2].

The outcomes of CFE are affected by several factors, such as patient comorbidities, the extent of arterial occlusion, and the existence of concurrent vascular diseases. Early postoperative complications and long-term patency rates significantly influence patient prognosis after CFE, as indicated by research. Current literature indicates that CFE can provide symptomatic relief and enhance the quality of life; however, careful patient selection and stringent follow-up protocols are crucial for optimising successful outcomes [3].

This study synthesises recent findings on the outcomes of CFE, evaluating its efficacy, complications, and predictors of success in patients undergoing the procedure.

## Materials and methods

Study design and population

This study is a descriptive retrospective analysis evaluating the outcomes of CFE with profundoplasty.

Inclusion Criteria

This study examined all patients with chronic limb-threatening ischaemia (CLTI) who underwent CFE with profundoplasty, with or without aortoiliac intervention, at Glan Clwyd Hospital (Wales, United Kingdom).

Exclusion Criteria

Patients who underwent bypass surgery involving the CFE were excluded from the study.

Data collection

Data were collected from patients who underwent CFE at Glan Clwyd Hospital, from January 2021 to January 2024. The clinical evaluation encompassed variables such as patient age, gender, smoking status, comorbidities, complications, primary patency, limb salvage, and mortality rates.

Follow-up

The follow-up was done in the vascular lab and assessed by Doppler US after six months of surgery to assess patency and then reviewed in an outpatient clinic.

Data analysis

The data for this study were processed using Statistical Product and Service Solutions (SPSS, version 21.0; IBM SPSS Statistics for Windows, Armonk, NY) software, encompassing data entry, cleaning, and analysis. Descriptive statistics were employed to display frequency tables along with their associated percentages. The means and standard deviations were reported as well. The chi-square test was utilised for categorical variables. A significance level of 0.05 or lower was deemed statistically significant, suggesting a meaningful relationship between the variables.

Quality assurance

To ensure the accuracy and reliability of the data, the following quality assurance measures were performed: Data collection was standardised using a uniform datasheet to minimise variability and errors. Four researchers independently reviewed the clinical and patient data to ensure consistency and avoid errors. An experienced biostatistician performed statistical analysis to ensure the validity of the results.

## Results

Seventy-seven patients were included over a three-year period. Of the participants, 46 (59.7%) were male, and 31 (40.3%) were female, resulting in a male-to-female ratio of 1.5:1.

The average age was 73.3 years with a standard deviation of 7.8 years. Hypertension and smoking were prevalent comorbidities among the majority of patients, whereas other comorbidities occurred less frequently (see Table 1).

**Table 1:** Patient demographics and comorbidities. CKD: Chronic Kidney Disease; COPD: Chronic Obstructive Pulmonary Disease

Variable	Present or Absent	Number	Percentage
Stroke	Yes	5	6.5%
	No	72	93.5%
CKD	Yes	10	13%
	No	67	87%
Hypertension	Yes	57	74%
	No	20	26%
Diabetes	Yes	24	31.2%
	No	53	68.8%
Cardiac diseases	yes	32	41.6%
	No	45	58.4%
Smoking	yes	57	74%
	No	20	26%
COPD	yes	9	11.7%
	No	68	88.3%
Total		77	100%

A majority of patients exhibit rest pain, accounting for 70.1% (n = 54), while the remainder experience both rest pain and tissue loss 29.9% (n=23). Forty patients (51.9%) underwent CFE during the hybrid operation involving iliac angioplasty; conversely, 37 patients (48.1%) had isolated CFE.

The most frequently reported postoperative complications included groin infection, hospital-acquired pneumonia, and thrombosis, while the remaining complications were reported less frequently (see Table 2). The limb salvage rate was 74% (n = 57), and the mean primary patency rate was 10.7 ± 8.4 months SD.

**Table 2:** Complications and mortality. HAP: Hospital Acquired Pneumonia; AKI: Acute Kidney Injury

Variable	Present or Absent	Number	Percent
Groin infection	yes	9	11.7%
	No	68	88.3%
Bleeding	yes	4	5.2%
	No	73	94.8%
HAP	yes	9	11.7%
	No	68	88.3%
AKI	yes	4	5.2%
	No	73	94.8%
Thrombosis	yes	9	11.7%
	No	68	88.3%
Mortality	yes	21	27.3%
	No	56	72.7%
Total		77	100%

The cross-tabulation test was conducted to evaluate factors influencing the limb salvage rate. Hypertension, cardiac disease, and chronic kidney disease are linked to a heightened risk of major amputation, with P values of 0.04, 0.02, and 0.039, respectively.

## Discussion

Despite recent advancements in endovascular devices, common femoral artery endarterectomy (CFE) remains the standard procedure for addressing CFA disease [4-7]. The endovascular treatment of the CFA region exhibits a significant recurrence rate, necessitating secondary intervention [8,9]. Bath et al. conducted a recent systematic review indicating that endovascular intervention is more advantageous for a specific subset of patients [10].

Nine patients (11.7%) were reported to have a groin infection. Three patients underwent debridement in the operating room, while the remaining cases were managed conservatively with antibiotics, a rate that is slightly higher than what is reported in the literature. Siracuse et al. documented a 6.3% incidence of groin infections in their series [11]. Nguyen et al. reported a gross infection rate of 8% in their series [12]. Elsherif et al. reported a 12% incidence of wound infection, consistent with our findings [13]. The observed discrepancies are likely associated with sample size, patient age groups, and body mass index (BMI). All patients underwent bovine patch closure; thus, we could not associate the infection with the type of patch closure used. The majority of cases involved the CFE in conjunction with iliac artery angioplasty to address inflow disease, while the rest of the cases had CFE as a standalone procedure. Several authors have documented favourable outcomes associated with CFE alone, noting acceptable results, minimal need for reintervention, and a high patency rate, consistent with our findings [14-17]. Acute kidney injury occurred in only four patients due to the contrast agent utilised for iliac disease treatment, and none required dialysis. Elsherif et al. documented acute kidney injury in 13% of the cases. This may result from the number of cases and the number of individuals who experienced the CFE in a hybrid operation [13]. The overall perioperative morbidity observed in this study was 28.6%. Nguyen et al. [12] documented a 15% incidence of combined morbidity and mortality factors. Elsherif et al. reported a perioperative mortality rate of 30% [13]. The observed discrepancies may be associated with the age group of our patients and their various comorbidities. The limb salvage rate was 74%, representing the lowest recorded in the literature.

A multicentre study involving 118 limbs reported a limb salvage rate of 82% at the five-year mark [4]. Additionally, other researchers documented a two-year limb salvage rate exceeding 90% [12,13]. The discrepancy in our limb salvage outcomes was attributed to the pattern of vascular diseases and their presentation, which occasionally complicated the management of inflow disease. The overall mortality rate during the three-year follow-up period was 27.3% among the patients (n = 21), with only two perioperative deaths (2.8%) attributed to major cardiovascular events (MACE). Kechagias et al., in a review of 111 limbs, reported a perioperative mortality rate of 1.8% [5]. In a single-centre study spanning eight years involving 117 patients, Ballotta et al. reported no instances of mortality [18]. The observed difference primarily pertains to the criteria used for patient selection in the intervention.

Study limitations

This study is a retrospective analysis of data collected from a single centre. Information regarding the clamping site, preoperative Ankle Brachial Index, patient symptom improvement, and postoperative wound healing was not documented.

## Conclusions

There is a reasonable patency rate associated with the common femoral endarterectomy, which is a possible operation that can be performed either independently or in conjunction with iliac angioplasty. Patients who are considered to be at high risk can be administered it. However, in order to prevent or reduce the severity of the postoperative issues, they are going to require a stringent follow-up appointment.

## References

[1] Crawford F, Welch K, Andras A, Chappell FM (2016). Ankle brachial index for the diagnosis of lower limb peripheral arterial disease. Cochrane Database Syst Rev.

[2] Conte MS, Bradbury AW, Kolh P (2019). Global vascular guidelines on the management of chronic limb-threatening ischemia. J Vasc Surg.

[3] Geale AT, Zayed H, Lamata P, Alastruey J, Clough RE (2024). Treatment of common femoral artery steno-occlusive disease: a comprehensive review of anatomical and hemodynamic considerations. J Cardiovasc Surg.

[4] Kuma S, Tanaka K, Ohmine T (2016). Clinical outcome of surgical endarterectomy for common femoral artery occlusive disease. Circ J.

[5] Kechagias A, Ylönen K, Biancari F (2008). Long-term outcome after isolated endarterectomy of the femoral bifurcation. World J Surg.

[6] Wieker CM, Schönefeld E, Osada N, Lührs C, Beneking R, Torsello G, Böckler D (2016). Results of common femoral artery thromboendarterectomy evaluation of a traditional surgical management in the endovascular era. J Vasc Surg.

[7] Kang JL, Patel VI, Conrad MF, Lamuraglia GM, Chung TK, Cambria RP (2008). Common femoral artery occlusive disease: contemporary results following surgical endarterectomy. J Vasc Surg.

[8] Derksen WJ, de Vries JP, Vink A (2010). Histologic atherosclerotic plaque characteristics are associated with restenosis rates after endarterectomy of the common and superficial femoral arteries. J Vasc Surg.

[9] Azéma L, Davaine JM, Guyomarch B, Chaillou P, Costargent A, Patra P, Gouëffic Y (2011). Endovascular repair of common femoral artery and concomitant arterial lesions. Eur J Vasc Endovasc Surg.

[10] Bath J, Avgerinos E (2016). A pooled analysis of common femoral and profunda femoris endovascular interventions. Vascular.

[11] Siracuse JJ, Gill HL, Schneider DB, Graham AR, Connolly PH, Jones DW, Meltzer AJ (2014). Assessing the perioperative safety of common femoral endarterectomy in the endovascular era. Vasc Endovascular Surg.

[12] Nguyen BN, Amdur RL, Abugideiri M, Rahbar R, Neville RF, Sidawy AN (2015). Postoperative complications after common femoral endarterectomy. J Vasc Surg.

[13] Elsherif M, Tawfick W, Elsharkawi M, Campell R, Hynes N, Sultan S (2018). Common femoral artery endarterectomy in the age of endovascular therapy. Vascular.

[14] Töpel I, Wiesner M, Uhl C, Betz T, Steinbauer MG (2015). Retrograde thrombendarterectomy vs. ilio-femoral bypass in unilateral iliac TASC C and D lesions. Vasa.

[15] Cardon A, Aillet S, Jarno P, Bensalah K, Le Du J, Idrissi A, Kerdiles Y (2001). [Endarteriectomy of femoral tripod: long-term results and analysis of predictive factors of failure]. Ann Chir.

[16] Al-Khoury G, Marone L, Chaer R (2009). Isolated femoral endarterectomy: impact of SFA TASC classification on recurrence of symptoms and need for additional intervention. J Vasc Surg.

[17] Desai M, Tsui J, Davis M, Myint F, Wilson A, Baker DM, Hamilton G (2011). Isolated endarterectomy of femoral bifurcation in critical limb ischemia: is restoration of inline flow essential?. Angiology.

[18] Ballotta E, Gruppo M, Mazzalai F, Da Giau G (2010). Common femoral artery endarterectomy for occlusive disease: an 8-year single-center prospective study. Surgery.

